# Supplementary material for: Characteristics of “chūnibyō” identified by a questionnaire
Source: PLoS One. 2021 Nov 24;16(11):e0260375. doi: 10.1371/journal.pone.0260375 (PMC8612564; doi:10.1371/journal.pone.0260375)
Supplement: Attachment — Submitted filename: Response to Reviewers.docx [file pone.0260375.s002.docx]

**Responses to the editor and the reviewers**

PONE-D-21-21951

“Characteristics of “chūnibyō” identified by a questionnaire”

**All modifications have been highlighted in the revised manuscript.**

To the editor and the reviewer:

Thank you very much for your constructive comments on our manuscript PONE-D-21-21951. We are pleased to hear that the reviewer found our study intriguing. In response to the reviewer’s requests and comments, we generated text. We carefully studied all comments and made the necessary edits/modifications. The revised version of the manuscript has been edited by a professional English-editing service. The point-by-point responses are listed below. We hope that we have sufficiently addressed the issues raised by the reviewer.

**Regarding the editor's comments:**

*I agree that it it worth considering the effects across cultures where it is known by different names. It is similar to the Messiah Complex that occurs in Christian societies and may also be related to the prophetic ideal.*

Response: Thank you very much for your comments and important introductions. It is very interesting, and we think that Messiah Complex is similar to Chūnibyō. However, Chūnibyō can generally occur during puberty. On the other hand, Messiah Complex may be not related to puberty. Therefore, we did not include Messiah Complex in our report.

**Regarding the reviewer's comments:**

*Reviewer #1: This article is very interesting and innovative. This fulfills its promise of demonstrating the characteristics of chūnibyō, something scholars have never comprehensively formulated. By using a mixed approach between quantitative and qualitative, this article is able to innovatively formulate the characteristics of chūnibyō. The working method is explained in a sequential manner and written in a clear and logical manner so that the results are very convincing. The results of this study will be an important reference for future research on similar topics. In addition, this article will provide inspiration to conduct research in the same field in the future (see below).*

Response: Thank you very much for your comments. It is very encouraging. We are glad you reviewed our manuscript. We modified the text based on your comment.

*-Minor suggestions are only given to the “Chūnibyō in other countries” subsection. This section is written with* *assumptions, without evidence and references, but the points presented are quite important and interesting. For this reason, it is recommended that this point be placed at the end of the article as a future topic research.* *It is a matter of facts, that Japanese culture has spread to various parts of the world, many children and teenagers are addicted to Japanese games or anime, and this means that negative impacts that are similar to the symptoms of Chūnibyō, may occur. If true, this is a good research topic because of its cross-country, cross-cultural, and cross-disciplinary characteristics.*

Response: Thank you very much for your helpful comments and important introductions. It’s exactly as your comment, so we modified it on page 28, lines 357-361.

**Journal requirements:**

*1. Please ensure that your manuscript meets PLOS ONE's style requirements, including those for file naming.*

Response: We confirmed that our manuscript met the PLOS ONE's style requirements.

*2. Please provide additional details regarding participant consent. In the Methods section, please ensure that you have specified (1) whether consent was informed and (2) what type you obtained (for instance, written or verbal). If your study included minors, state whether you obtained consent from parents or guardians. If the need for consent was waived by the ethics committee, please include this information.*

Response: Our manuscript demonstrated participant consent. Informed consent was obtained from all respondents in an anonymous online questionnaire, although signatures were not collected because the questionnaire was anonymous. We obtained participant consent through respondents selecting a checkbox indicating their intention to agree in an online questionnaire. This study protocol was approved by the Institutional Review Board of Fukujuji Hospital (Study number: 20064). We added type of an obtention for participant consent in our manuscript on page 7, lines 105-107.

*3. In your Data Availability statement, you have not specified where the minimal data set underlying the results described in your manuscript can be found. PLOS defines a study's minimal data set as the underlying data used to reach the conclusions drawn in the manuscript and any additional data required to replicate the reported study findings in their entirety. All PLOS journals require that the minimal data set be made fully available. For more information about our data policy, please see* [*http://journals.plos.org/plosone/s/data-availability*](http://journals.plos.org/plosone/s/data-availability)*.*

Response: We uploaded data availability statement of our study as supplemental file newly. Please confirm it.

*4. We note that you have indicated that data from this study are available upon request. PLOS only allows data to be available upon request if there are legal or ethical restrictions on sharing data publicly. For more information on unacceptable data access restrictions, please see http://journals.plos.org/plosone/s/data-availability#loc-unacceptable-data-access-restrictions.*

*In your revised cover letter, please address the following prompts:*

*a) If there are ethical or legal restrictions on sharing a de-identified data set, please explain them in detail (e.g., data contain potentially sensitive information, data are owned by a third-party organization, etc.) and who has imposed them (e.g., an ethics committee). Please also provide contact information for a data access committee, ethics committee, or other institutional body to which data requests may be sent.*

*b) If there are no restrictions, please upload the minimal anonymized data set necessary to replicate your study findings as either Supporting Information files or to a stable, public repository and provide us with the relevant URLs, DOIs, or accession numbers. For a list of acceptable repositories, please see http://journals.plos.org/plosone/s/data-availability#loc-recommended-repositories.*

*We will update your Data Availability statement on your behalf to reflect the information you provide.*

Response: Same as response to Q3, we uploaded data availability statement of our study as supplemental file newly. Please confirm it.

*5. Please upload a copy of Figure 1 of page 11 and 2 for page 21, to which you refer in your text. If the figure is no longer to be included as part of the submission please remove all reference to it within the text.*

Response: We uploaded them on the submission system.
